# The role of tracheal wall injury in the development of benign airway stenosis in rabbits

**DOI:** 10.1038/s41598-023-29483-2

**Published:** 2023-02-23

**Authors:** Jie Zhang, Yue hong Liu, Zhen yu Yang, Zi yi Liu, Chang guo Wang, Da xiong Zeng, Jun hong Jiang

**Affiliations:** 1 Department of Respiratory and Critical Care Medicine, Dushu Lake Hospital to Soochow University, Suzhou, 215125 Jiangsu China; 2 Department of Respiratory and Critical Care Medicine, The First Affiliated Hospital of Soochow University, Suzhou, 215006 Jiangsu China

**Keywords:** Diseases, Medical research, Pathogenesis

## Abstract

To investigate the role of tracheal wall injury in the development of benign airway stenosis in rabbits. Prospective study. We injured the tracheal walls of 28 New Zealand white rabbits using four different methods. Experimental group: Group A (n = 7, mild injury of tracheal mucosa by ordinary brush under bronchoscopy); Group B (n = 7, severe injury of tracheal mucosa by nylon brush under tracheotomy); Group C (n = 7, tracheal cartilage was injured by vascular clamp after tracheotomy); Group D (n = 7, the tracheal cartilage was injured with vascular forceps and the tracheal mucosa was injured with a nylon brush after tracheotomy). Bronchoscopy was performed on each experimental rabbit at 1, 2, 3 and 4 weeks after operation. High-resolution computed tomography (HRCT) and endobronchial optical coherence tomography (EB-OCT) were performed at 4 weeks, and the rabbits were sacrificed after the examination. Their gross and histological findings were comparatively determined whether the experimental rabbit stenosis was established. No airway stenosis was observed in group A. In group B, 28.57% of experimental rabbits developed tracheal stenosis (granulation tissue proliferation was observed in rabbits No. 2 and No. 6 at 1, 2 and 3 weeks after operation, and the tracheal scar contracture was observed in No.6 rabbit at 4 weeks after operation). Fourteen rabbits in group C and group D had tracheal stenosis caused by granulation tissue proliferation at 1, 2 and 3 weeks after operation. At the fourth week after operation, 71.43% of experimental rabbits had tracheal stenosis due to granulation tissue hyperplasia, 7.14% of experimental rabbits had tracheal stenosis due to scar contracture and granulation hyperplasia, and 21.43% of experimental rabbits had tracheal stenosis due to scar contracture. EB-OCT scan showed that the cartilage layer with low signal reflection band was discontinuous. The injury of cartilage is the key factor of benign airway stenosis. Acute injury of airway mucosa alone is unlikely to cause airway stenosis, but combined with cartilage injury may aggravate airway stenosis. EB-OCT can clearly identify the airway layers of rabbits, which is helpful to evaluate the damage of tracheal cartilage and mucosa. The diagnostic potential of this technique makes EB-OCT a promising approach for the study and monitoring of airway diseases.

## Introduction

Benign airway stenosis is caused by various non-malignant tumor lesions, including tuberculosis, benign airway tumors, laryngomalacia or tracheomalacia, and iatrogenic laryngotracheal stenosis (iLTS). In the past, tuberculosis was considered the leading cause of benign airway stenosis in China^1^. In recent years, some retrospective studies in China have found that iatrogenic injuries such as tracheal intubation and tracheostomy are the primary causes of benign airway stenosis in China^2^. Airway stenosis is also the most common complication after tracheal intubation or tracheotomy, occurring in 10% to 22%^3^. The pathogenesis of airway stenosis is complex. Hillel et al.^4^ showed that epithelium plays an important role in the formation of idiopathic subglottic stenosis (iSGS) and iatrogenic laryngotracheal stenosis. Increasing evidence supports that iLTS is an active fibroinflammatory response mediated by dysregulated host immunity^5^. Specifically, M2 macrophages and Th2 cellshave been implicated in the pathophysiology of iLTS^5–8^, upregulation of immune checkpoints, programmed cell death protein 1 (PD-1) receptor (CD279) and its major protein ligands, PD-L1 (B7-H1; CD274), binding to CD4 T cells has also recently been observed in LTS patients, and TGFβ1 (transforming growth factor β1) has been identified as a potential mechanism of PD-L1 upregulation on fibroblasts in iLTS^9^. These immune alterations ultimately lead to fibroblast activation and increased extracellular matrix deposition, which results in airway stenosis^5,10,11^. A number of methods for inducing airway stenosis in animal models have been described, including brushing, thermal ablation damage, chemical damage, endotracheal intubation, and partial cartilage ring excision^12–18^. Trauma is an important cause of benign airway stenosis. However, the degree of airway wall injury in the formation of benign airway stenosis needs to be further explore. In this study, we used different methods to damage the tracheal walls of rabbits, and explored the role of airway wall injury in the formation of benign airway stenosis in rabbits.

## Materials and methods

### Different experimental groups

Twenty-eight 4-month-old New Zealand white rabbits, weighing 2.5–3.5 kg, from Suzhou Huqiao Biotechnology Co., LTD. (Suzhou, China), were randomly divided into four groups (7 rabbits/group): A, B, C, and D. Random numbers were generated using the standard = RAND() function in Microsoft Excel. All protocols in this study were approved by the Committee on the Ethics of Animal Experiments of Dushu lake hospital to Soochow University Animal, Suzhou, China (IACUC permit number: 220048), in compliance with the Guide for the Care and Use of Laboratory Animals published by the US National Institutes of Health (NIH publication no.85-23, revised 1996). All methods were carried out in accordance with the ARRIVE guidelines and regulations. We employed four different tracheal injury methods, and rabbits were groups as follows: Group A (n = 7), tracheal mucosa was slightly damaged by an ordinary brush through tracheoscopy; Group B (n = 7), tracheal mucosa was severely damaged by a nylon brush after tracheotomy; Group C (n = 7), tracheal cartilage was injured with a vascular clamp after tracheotomy; and Group D (n = 7), tracheal mucosa was injured with a nylon brush and the tracheal cartilage was damaged by a vascular clamp. Severe mucosal injury was defined as obvious mucosal floating when the brush was immersed in normal saline after use.

### Methods of tracheal injury (Table 1)

**Table 1:** Interventions for experimental rabbits in each group.

Group	Tracheal mucosa damage	Tracheal cartilage damage
A	+	−
B	++	−
C	−	++
D	++	++

“ + ” indicates that the measure was applied to the experimental animals; “–” indicates that the animals were not treated with the measure.

**Figure 1:**
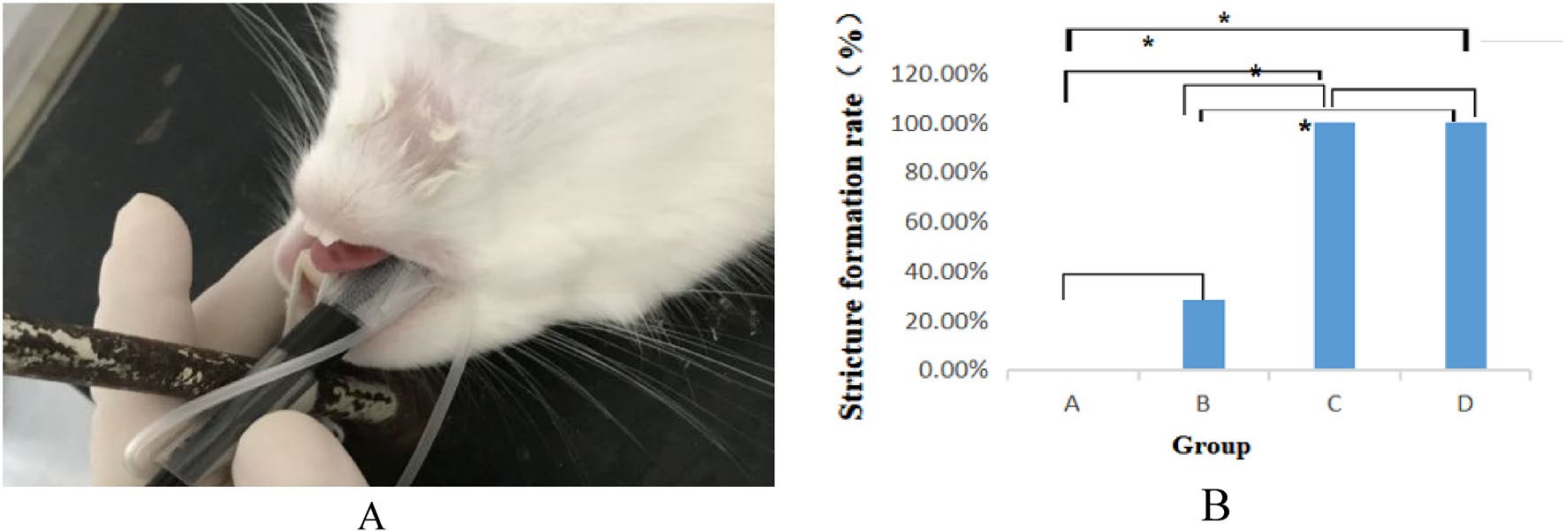
(**A**) Tracheal intubation in a rabbit; (**B**) comparison of the incidence of stenosis. *p < 0.05,using Fisher's exact test.

All rabbits were fasted for 6 h before surgery, intramuscularly anesthetized with 0.1 mL/kg of xylazine hydrochloride, and placed in the supine position on the operating table. After anesthesia, we intubated group A rabbits to the glottis using an endotracheal tube with an inner diameter of 6.5 mm in order to avoid destruction of the bronchoscope by the rabbit molars (Fig. 1A). An ordinary brush with a diameter of 5.5 mm was then inserted into the trachea through a bronchoscope, and the tracheal mucosa was circumferentially scraped by pushing and pulling the brush 20 times. The anterior neck of each rabbit in groups B, C, and D was shaved and sterilized. After a midline skin incision was made in the anterior neck, the larynx and trachea were exposed, taking care not to injure the sternohyoid and thoracothyroid muscles. Starting 1.5 cm from the caudal margin of the cricoid cartilage, the trachea was cut along the smooth muscle and elastic fibers between the cartilage rings, and the incision was 2/3 of the circumference of the trachea. In Group B, a nylon brush with a diameter of 5.5 mm was inserted into the trachea via the tracheal incision, and the tracheal mucosa was then circumferentially scraped by pushing and pulling the brush 20 times. In group C, 2 cricoid cartilages on both sides of the tracheal incision were destroyed with vascular forceps. In group D, the tracheal mucosa was scraped by the same method as that in group B, and the tracheal cartilage was destroyed by the same method as that in group C. After the operation, the brush was immersed in normal saline, and obvious mucosal floating was observed in groups B and D. All rabbits in Group B, C, and D were examined for bleeding in the trachea, and if necessary, gauze compression hemostasis was applied. After ensuring the vital signs of rabbits in all groups were stable, the trachea incision, subcutaneous tissue, and skin were sutured. All rabbits were intramuscularly awakened with 0.1 mL/kg Luxingning II after surgery.

### Follow-up examination at different time points

The formation of airway stenosis was observed by bronchoscopy at 1, 2, 3 and 4 weeks after operation. At week 4, all experimental rabbits underwent bronchoscopy, endobronchial optical coherence tomography (EB-OCT) and computed tomography (CT). At the end of the examination, the experimental rabbits were sacrificed by air embolization, and larynx and trachea specimens were obtained. The specimens were fixed in 10% buffered formalin, and some fresh tracheal specimens were placed in a freezer at −80 °C.

### Airway stenosis was evaluated by CT

Thin-slice scans were performed on a Philips 64-row 128-slice CT (Philips, Amsterdam, The Netherlands). The scanning parameters were as follows: layer acquisition thickness: 1 mm, tube voltage: 120 kV, and tube current: 78 mA. The inner diameter of the trachea was measured in a fat window (window width 500 Hu, window level −100 Hu)^19^. The degree of tracheal stenosis was evaluated using the following two indexes: the transverse diameter, and the longitudinal diameter of the trachea. The former index (%) is defined as^20^:

$${\text{S}}\, = \,\left[ {1 - \, \left( {{\text{d}}1 \times {\text{d}}2} \right)/\left( {{\text{D}}1 \times {\text{D}}2} \right)} \right]\, \times \,100\% ,$$

where d1 and d2 are the transverse and longitudinal diameters of the narrowest lumen, respectively, and D1 and D2 are the transverse and longitudinal diameters 1 cm below the narrowest segment, respectively. According to no stenosis, ≤ 25%, 26–50%, 51–75%, 76%-90%, 90% to complete obstruction, the degree of stenosis in animals was divided into 0, 1,2, 3, 4, and 5, respectively^21^.

### Data analysis

Results statistical analysis was performed using SPSS 23.0 software (IBM Corporation, Armonk, New York, USA). Fisher's exact test was used to compare stricture formation rates between groups. The degree of stenosis was compared using the Mann–Whitney. A *P*-value < 0.05 was considered statistically significant.

### Ethics approval and consent to participate

Obtained from the Medical Ethics Committee of Dushu lake hospital to Soochow University (220048).

## Results

**Figure 2:**
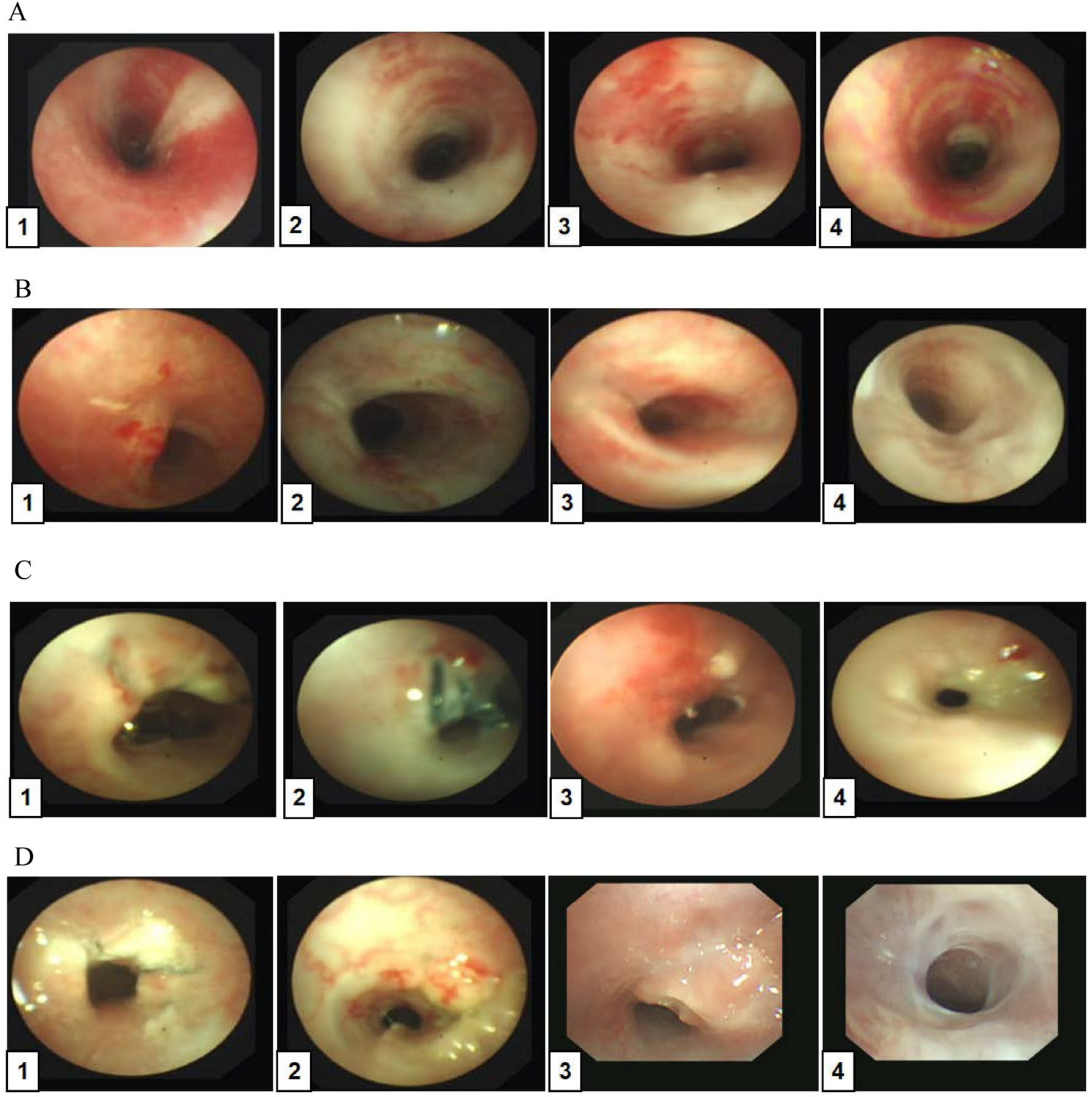
Follow-up pictures of experimental rabbits under bronchoscopy. (**A–D**) The follow-up pictures of experimental rabbits in group A, B, C and D, respectively.

**Figure 3:**
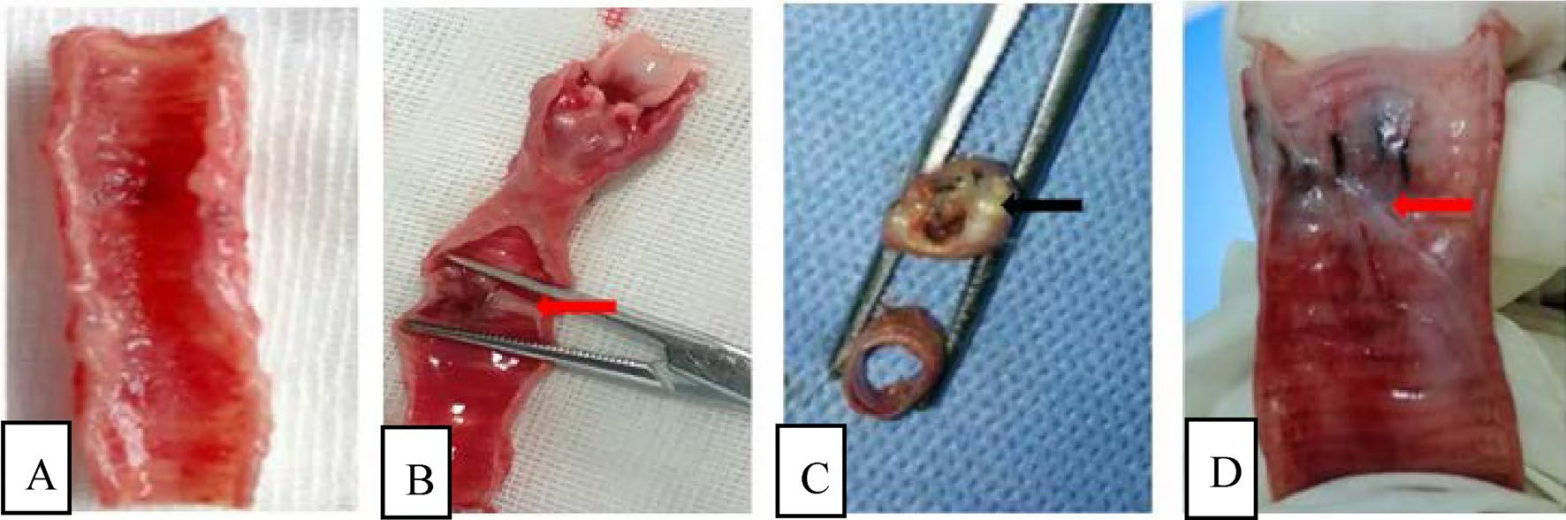
Examination of a gross specimen. (**A–D**) The gross anatomy of experimental rabbits in Groups A, B, C, and D, respectively. Images (**B,D**) shows scar contracture (red arrow); Image (**C**) show granulation tissue proliferating (black arrow) with yellowish-white secretions attached to the surface.

**Figure 4:**
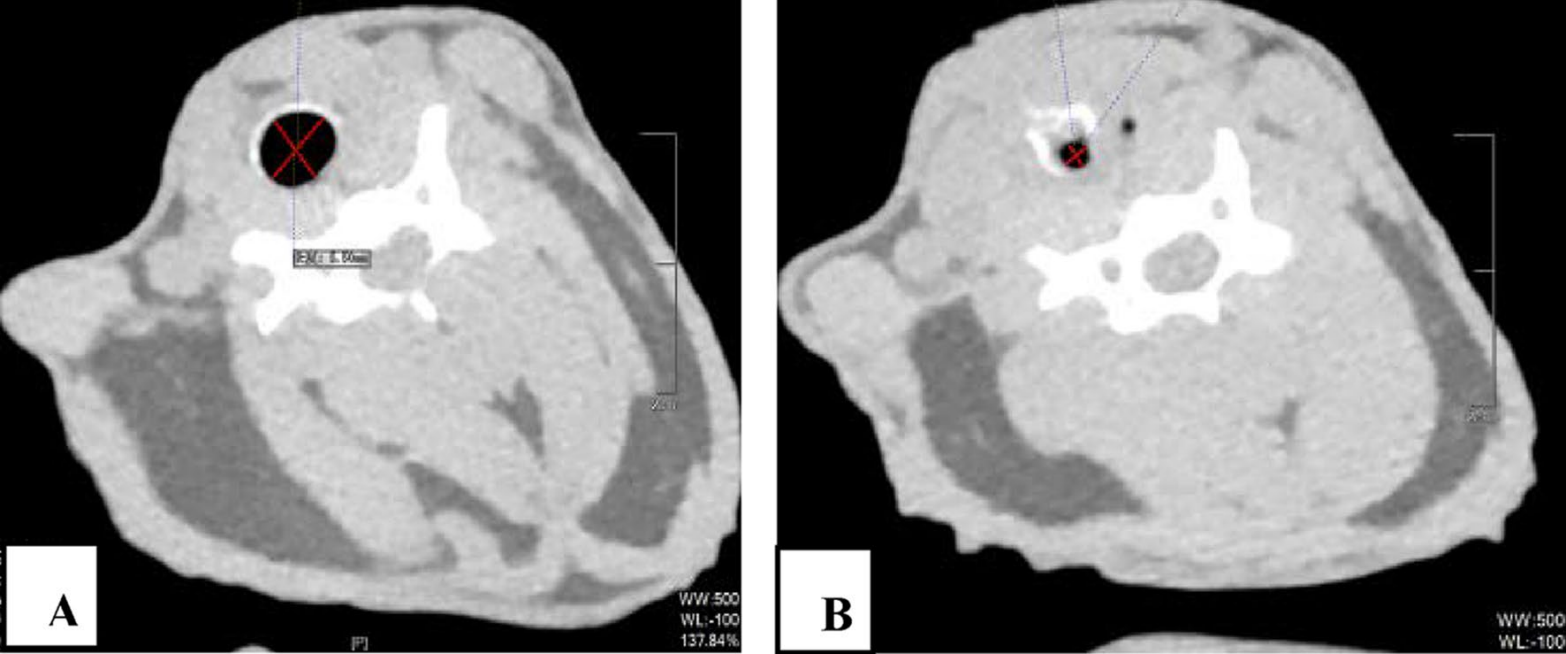
CT scans of rabbits with tracheal stenosis. The CT diagram of the internal tracheal diameter measured by the same experimental rabbit fat window (window width 500 Hu, window level −100 Hu): (**A**) normal tracheal lumen, (**B**) narrow tracheal lumen. *CT* computed tomography.

**Figure 5:**
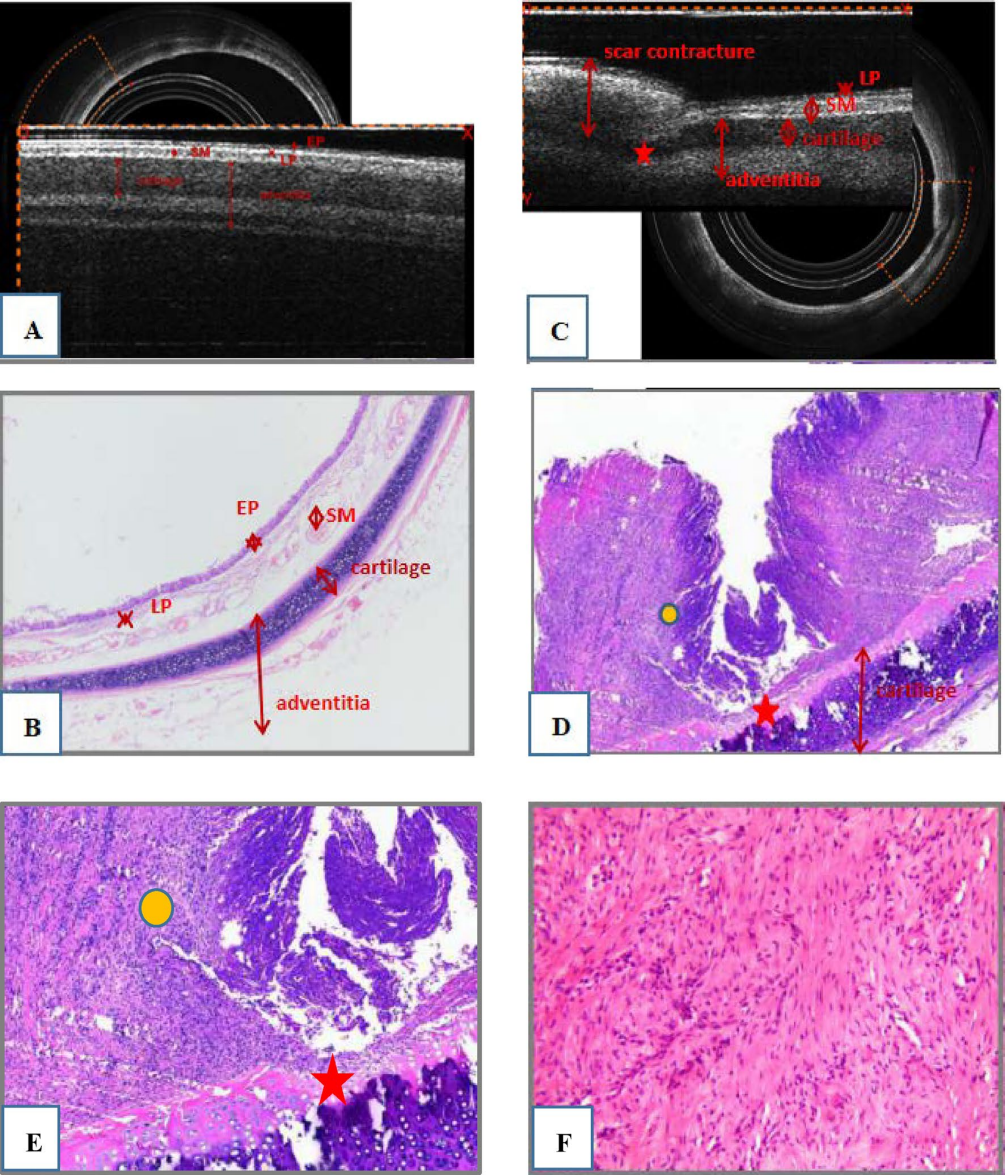
(**A**) EB-OCT images of rabbits without airway stenosis, (**B**) airway histological characteristics of rabbits without airway stenosis (HE; original magnification ×40). (**C**) EB-OCT images of rabbits with airway stenosis, (**D,E**) airway histologic characteristics of rabbits with airway stenosis. D (HE; original magnification ×100) and (**E**) (HE; original magnification ×200) airway stenosis showing ciliated columnar epithelial necrosis and shedding, a small amount of residual epithelial tissue, the submucosal fibrous tissue proliferating and protruding into the tracheal lumen, inflammatory cell infiltration in the interstitium (yellow circle), the smooth muscle of the tube wall is broken and atrophied, and the focal cartilage is damaged (red pentagram); (**F**) fibroblastic proliferation *EB-OCT* endobronchial optical coherence tomography, *HE* hematoxylin–eosin, *EP* epithelial, *LP* laminae propria, *SM* submucosa.

1. Comparison of the incidence of stenosis (Fig. 1B)
  The total tracheal stenosis rate in the four groups was 57.14% (16/28). The airway stenosis rates of group A, B, C and D were 0.00%, 28.57%, 100% and 100%, respectively. The formation rate of airway stenosis in Groups C and D was higher than that in Groups A and B. Groups C and D were significantly different from Groups A and B (*P* < 0.05), and there was no difference between Groups A and B (*P* > 0.05). The degree of stenosis in Groups C and D was 16.59–76.29% and 50.59–87.42%, respectively. There was no difference between Groups C and D (*P* > 0.05).
2. Bronchoscopic manifestations of experimental rabbits (Fig. 2)
  In group A, no airway stenosis was observed during the follow-up of bronchoscopy for 4 weeks (Fig. 2A). This was also confirmed by tracheal anatomy (Fig. 3A). In group B, granulation tissue was observed in rabbits No. 2 and No. 6 at 1 week after operation. At 2, 3, and 4 weeks after operation, the granulation tissue gradually increased, and the lumen stenosis further increased in No. 2 rabbit. Scar contracture was observed at 4 weeks after operation in No. 6 rabbit (Fig. 2B). These are also consistent with tracheal anatomy (Fig. 3B). During the 4-week follow-up period, no stenosis was observed in the remaining five rabbits in group B. After tracheoscopy at week 4, one rabbit in group B died and pulmonary edema was found by autopsy.The proliferation of granulation tissue was observed in the tracheal lumen of 14 rabbits in group C and group D, and the granulation tissue gradually increased 2 and 3 weeks after operation. At 4 weeks after operation, granulation tissue hyperplasia and scar contracture were observed in the tracheal lumen of rabbits(Fig. 2C,D).This result was consistent with the tracheal anatomy(Fig. 3C,D).
3. The CT performance of the experimental rabbits (see Fig. 4A,B)
  CT scan of rabbits in group A showed no tracheal stenosis. In group B, CT scan of rabbit No. 2 and No. 6 showed tracheal stenosis. CT scan of 14 rabbits in group C and group D showed tracheal stenosis.
4. Image characteristics of EB-OCT scans
  EB-OCT scan results of rabbits without airway stenosis in group A and group B were stratified gray scale scans according to signal intensity, respectively. In EB-OCT scanning, the mucosa showed weak signals in gray, the submucosa showed strong signals in white, the smooth muscle layer showed grayish-white signal intensity, and the cartilage layer showed weak signals in black (see Fig. 5A). EB-OCT images of experimental rabbits with tracheal stenosis showed obvious stenosis of the lumen, thickening of the mucosa and submucosa (strong signal reflection band), and some layers were projected inward, and the cartilage layer was discontinuous (indicated by low signal reflection band) (see Fig. 5C).
5. Pathological results
  In the rabbits without tracheal stenosis, the tracheal mucosal surface showed pseudostratified ciliated columnar epithelium, a small amount of inflammatory cell infiltration in the submucosa, and the perichondrium and cartilage structure were intact (see Fig. 5B). In the experimental rabbits with tracheal stenosis, mucosal necrosis and exfoliation, infiltration of a large number of inflammatory cells in the mucosa and submucosa, interstitial congestion and edema, proliferation of submucosal capillaries, formation of granulation tissue, proliferation of fibroblasts, and destruction of cartilage structure were observed (see Fig. 5D–F).

## Discussion

The New Zealand white rabbit is the preferred model for the study of human tracheal stenosis, as the rabbit larynx corresponds closely to that of humans^22^. Moreover, the rabbit airway is close to the size and complexity of a human newborn^23,24^. Similar to the human trachea, the rabbit trachea wall is composed of mucosa, submucosa, and adventitia. The mucosa layer includes pseudostratified ciliated columnar epithelium and lamina propria, and the submucosa contains loose connective tissue with serous glands. The adventitial layer is composed of a C-type hyaline cartilage ring and dense connective tissue. The cartilage rings are connected by smooth muscle and elastic fibers. In recent years, with the development of Ultra-thin bronchoscopy, the difficulty of interventional operation in rabbit airway has been reduced. Therefore, we chose healthy New Zealand white rabbits to explore the role of airway wall damage in the formation of benign airway stenosis. At the same time, it also provides the possibility for the interventional treatment of benign airway stenosis animal model.

The concept of OCT was originally developed by D. Huang and J. G. Fujimoto et al. at the Massachusetts Institute of Technology in the United States Proposed in 1991^25^. In 2004, Jung et al.^26^ used OCT to scan the trachea of normal rabbits and the trachea of septic rabbits, and compared the results with histopathology. The results showed that OCT could detect the hierarchical structure of the trachea in detail, including the epithelial layer, mucosa layer, submucosa layer and cartilage layer. The morphological structure of OCT images was similar to that of pathological images. In 2009, Williamson et al.^27^ performed OCT scanning of the human airway and compared it with histopathology. The results showed that OCT could display the images of bronchial epithelial layer, mucosal layer, cartilage layer and glands, which was highly consistent with histological examination. Therefore, we used EB-OCT to visualize the rabbit tracheal.

We used different methods to injure the airway wall of rabbits in different degrees, and the airway stenosis was observed by bronchoscopy, HRCT and EB-OCT. The formation of tracheal stenosis observed by HRCT and EB-OCT was consistent with the results of bronchoscopy and tracheal anatomy. The EB-OCT images of rabbits without tracheal stenosis showed gray-scale images, in which the mucosa showed weak gray signal, the submucosa showed strong white signal, the smooth muscle layer showed gray signal intensity, and the cartilage layer showed weak black signal. The EB-OCT images of rabbits with tracheal stenosis formation showed obvious narrowing of the lumen, thickening of the mucosa and submucosa (strong signal reflection band), some layers projecting inward, and discontinuity of the cartilage layer (low signal reflection band). In group B, the tracheal wall of the 5 rabbits without tracheal stenosis was cut and sutured along the smooth muscle and elastic fibers between the cricoid cartilage, but no stenosis was found by bronchoscopy, HRCT, EB-OCT and tracheal anatomy. It can be seen that simple tracheotomy and incision suture do not cause stenosis of the rabbit tracheal. In group A and group B, the tracheal mucosa was injured with a brush. The postoperative pathology showed that the mucosal surface of the rabbits without tracheal stenosis was pseudostratified ciliated columnar epithelium, and the cartilage structure was intact, indicating that the tracheal mucosa could regenerate after simple acute injury, and there was no stenosis formation with the repair of the mucosa. The histopathological results of rabbits with tracheal stenosis showed that when the tracheal wall was damaged to the cartilage layer, the ability of mucosal regeneration was poor, a large number of inflammatory cells infiltrated in the submucosa, capillaries increased, granulation tissue formed, and fibroblasts proliferated. EB-OCT and tracheal histopathology showed that cartilage injury was a key factor in the formation of benign tracheal stenosis. This is consistent with the results of previous studies^28–30^.

The degree of stenosis in group C and group D were 16.59–76.29% and 50.59–87.42%, respectively. Although there was no significant difference between the two groups, the proportion of grade 3 or above stenosis was 42.86% in group C and 100% in group D. This suggests that the formation of tracheal stenosis may be related to the function of tracheal mucosa. An intact tracheal mucociliary muco-system barrier removes exogenous stimuli; however, mucosal injury reduces clearance capacity. A mouse model of airway obstruction has shown that airway epithelial cells can regulate the growth of fibroblasts and prevent the overgrowth of fibroblasts from causing lumen obstruction^31^. Hillel et al.^4^ showed that compared with non-scar epithelium, the epithelium within iSGS and iLTS is morphologically abnormal. Although both iSGS and iLTS have reduced epithelial thickness, ciliary cells, and secretory cells, only iSGS had signifificant increases in pathological basal cell expression.This suggests that the epithelium plays an important role in the pathogenesis of both types of stenotic fibrosis. In this experiment, the rabbit tracheal mucosa can regenerate after acute injury alone, but the ability of mucosal regeneration is weakened when there is both cartilage and mucosal injury. Chronic, persistent damage to the mucosa involving the cartilage may result in airway stenosis.

The tracheal stenosis was mainly caused by granulation tissue hyperplasia at 1 week after operation, and scar contracture was observed in some rabbits at 4 weeks after operation. This was also confirmed by gross anatomy. This suggests that we can choose different types of stenosis according to different time points for animal studies of benign airway stenosis, and provide different types of stenosis animal models for the study of interventional therapy of benign airway stenosis.

In conclusion, cartilage injury is the key factor in the formation of benign airway stenosis in rabbits. Mucosa plays a certain role in the formation of airway stenosis, but acute injury of airway mucosa alone is unlikely to cause airway stenosis, and combined with cartilage injury may aggravate the degree of airway stenosis. Chronic, persistent damage to the mucosa with involvement of cartilage can cause airway stenosis.

## Data Availability

The data that support the findings of this study are available from the corresponding author upon reasonable request.

## Abbreviations

HRCT: High-resolution computed tomography
EB-OCT: Endobronchial optical coherence tomography
iLTS: Iatrogenic laryngotracheal stenosis
CT: Computed tomography
iSGS: Idiopathic subglottic stenosis
PD-1: Programmed cell death protein 1
TGFβ1: Transforming growth factor β1
